# Impact of measles supplementary immunisation activities on utilisation of maternal and child health services in low-income and middle-income countries

**DOI:** 10.1136/bmjgh-2017-000466

**Published:** 2018-05-07

**Authors:** Iryna Postolovska, Stéphane Helleringer, Margaret E Kruk, Stéphane Verguet

**Affiliations:** 1 Department of Global Health and Population, Harvard T.H. Chan School of Public Health, Boston, Massachusetts, USA; 2 Department of Population, Family and Reproductive Health, Johns Hopkins University Bloomberg School of Public Health, Baltimore, Maryland, USA

**Keywords:** child health, immunisation, health systems, measles

## Abstract

**Background:**

Measles supplementary immunisation activities (SIAs) are an integral component of measles elimination in low-income and middle-income countries (LMICs). Despite their success in increasing vaccination coverage, there are concerns about their negative consequences on routine services. Few studies have conducted quantitative assessments of SIA impact on utilisation of health services.

**Methods:**

We analysed the impact of SIAs on utilisation of selected maternal and child health services using Demographic and Health Surveys and Multiple Indicator Cluster Surveys from 28 LMICs, where at least one SIA occurred over 2000–2014. Logistic regressions were conducted to investigate the association between SIAs and utilisation of the following services: facility delivery, postnatal care and outpatient sick child care (for fever, diarrhoea, cough).

**Results:**

SIAs do not appear to significantly impact utilisation of maternal and child services. We find a reduction in care-seeking for treatment of child cough (OR 0.67; 95% CI 0.48 to 0.95); and a few significant effects at the country level, suggesting the need for further investigation of the idiosyncratic effects of SIAs in each country.

**Conclusion:**

The paper contributes to the debate on vertical versus horizontal programmes to ensure universal access to vaccination. Measles SIAs do not seem to affect care-seeking for critical conditions.

**Key questions**

**What is already known?**

- Concerns exist about the potential negative consequences of measles supplementary immunisation activities (SIAs) on routine health services in low-income and middle-income countries (LMICs).

**What are the new findings?**

- We analysed the impact of SIAs on utilisation of selected maternal and child health services (maternal delivery, care-seeking for sick child) using Demographic and Health Surveys and Multiple Indicator Cluster Surveys from LMICs; and found that SIAs did not appear to significantly impact care-seeking for those services.

**What do the new findings imply?**

- We point to the need for further investigation of the idiosyncratic effects of SIAs in each country where they take place.
- The paper contributes to the debate on vertical versus horizontal programmes to ensure universal access to vaccination.

## Introduction

Measles continues to be a key contributor to child mortality, particularly in South Asia and sub-Saharan Africa. Despite recent declines in mortality, an estimated 115 000 deaths globally were attributed to measles in 2014, with most of the deaths occurring among children under 5 years.^1^ In order to reduce global measles mortality, WHO recommends reaching all children with two doses of measles vaccine.^2^

In most low-income and middle-income countries (LMICs), vaccines, including the first dose of measles vaccine, are primarily delivered through routine immunisation services at health facilities, but the majority of LMICs also conduct supplementary immunisation activities (SIAs), in particular for the second dose of measles vaccine, to ensure high coverage.^1 2^ Measles SIAs are mass campaigns during which health workers offer immunisations to children at fixed (permanent or temporary) or mobile vaccination posts. They occur every 2–4 years and often include the delivery of other child interventions, such as oral polio vaccine, vitamin A supplements, deworming medicines and insecticide-treated bed nets.^3–5^ The immunisation posts typically employ at least two health workers trained in injection techniques to administer the vaccine and 3–4 volunteers with no health training (responsible for recording, screening children and crowd control). The duration of SIAs depends on the number of children and the number of health workers available to deliver vaccines.^6^ On average, measles SIAs last approximately 16 days, but this varies substantially, ranging from <4 days in Malawi and Uganda to >40 days in Tunisia and Vietnam.^7^

First introduced in the 1990s, SIAs have contributed to measles elimination in the Americas^8^ and to recent reductions in measles incidence and mortality in sub-Saharan Africa.^9–11^ Several studies have documented the success of SIAs in improving coverage and equity of vaccination compared with routine immunisation,^12 13^ but the evidence remains mixed and limited in terms of the broader impact of SIAs on routine health services and primary healthcare.^14–16^ In areas with limited access to health services, SIAs might be able to reach individuals who would otherwise have limited contact with the health system. For example, in some areas of Angola and Rwanda, Closser *et al* 15 found that polio campaigns provided individuals an opportunity for face-to-face communication with health providers about routine immunisation and other health services.

As with any vertical programme, however, there are concerns that SIAs might exacerbate the shortage of health workers at facilities before and during the campaign and may thus adversely affect routine health services.^17–21^ Findings from qualitative studies have indeed shown that mass campaigns can result in health worker absence, interruption of services at health facilities and providers skipping important non-immunisation tasks, particularly in countries with weaker health systems.^14 15 22 23^

To date, few studies have conducted quantitative assessments of the impact of SIAs on the utilisation of routine health services in LMICs. For example, Verguet *et al* 24 examined the impact of the 2010 measles SIA in South Africa and found significant reductions in selected routine health services during the month the SIA was implemented. A recent study evaluating the impact of mass campaigns in Cameroon reported similar findings for outpatient visits and antenatal care consultations, with the effect more pronounced for ‘intensive’ campaigns—those lasting 7 days or less.^25^

In this study, we analysed the potential impact of measles SIAs on the utilisation of selected maternal and child health services using data from the Demographic and Health Surveys (DHS) and Multiple Indicator Cluster Surveys (MICS) in 28 LMICs. Specifically, this paper examined the potential SIA impact on maternal delivery services and care-seeking for sick child, as they are critical routine health services and indicators in LMICs.

## Methods

### Data sources

#### Supplementary immunisation activities

Information on all measles SIAs implemented between 2000 and 2014 was obtained from the WHO Immunization, Vaccines and Biologicals (IVB) database.^7^ This included the dates of campaign implementation and whether the campaign was regional or national. According to the WHO Measles SIA Planning and Implementation Field Guide, national campaigns are conducted simultaneously in all regions.^6^ If a national campaign is not feasible, it is recommended that countries adopt a ‘rolling’ approach by conducting the SIA in phases or target high-risk regions (subnational campaigns). Since information on the exact regions in which the subnational or rollover national campaigns were implemented was not available, they were excluded from our analysis. Therefore, our analysis focused on national SIAs and their potential impact on a country as a whole.

Between 2000 and 2014, 417 national measles SIAs were conducted in 132 countries, of which 45 countries were in the WHO African region, 27 in the Americas region, 17 in the Eastern Mediterranean region, 16 in the European region, 7 in the South-East Asia region and 20 in the Western Pacific region (table 1). The full list of countries and dates of national measles SIA campaigns is available from the WHO IVB database.^7^

**Table 1:** Number of measles supplementary immunisation activities (SIAs) implemented by WHO region over 2000–2014

WHO region	Number of measles SIAs		
	National campaigns	Rollover national campaigns	Subnational campaigns
African region	153	44	116
Region of the Americas	121	5	27
Eastern Mediterranean region	42	34	85
European region	34	9	18
South-East Asia region	13	26	20
Western Pacific region	54	12	46
Total	417	130	312

Source: WHO Immunization, Vaccines and Biologicals (IVB) Database.

#### MICS and DHS surveys

We identified all MICS and DHS conducted in countries within a year of the national measles SIAs to ensure an overlap with the dates of SIAs.^26 27^ The MICS and DHS are large, nationally representative household-based surveys conducted every 3–6 years. The two surveys are comparable, with some minor differences discussed in more detail further. Both have a similar clustered sampling design, with enumeration areas constituting the primary sampling unit and households constituting the final unit. The surveys sample women between the ages of 15–49 years and collect information on maternal and child health.^28–30^ Importantly for this analysis, the surveys provided dates of when the interviews were conducted and collected birth dates (eg, date of delivery). This allowed us to map the dates of care-seeking to the dates of SIA implementation, as discussed in more detail in the ’Analysis' subsection below.

For the purposes of this analysis, we limited the sample to low-income and lower-middle-income countries (excluding upper-middle-income countries), as the majority of SIAs were conducted in such settings, and these countries are also more prone to health worker shortages.^31 32^ Our analysis included 28 low-income and lower-middle-income countries that had conducted at least one SIA between 2000 and 2014 and had DHS and/or MICS data available. The list of countries and surveys is presented in table 2. On average, measles SIAs were conducted for 13 days in these countries (see online supplementary appendix figure A1).

10.1136/bmjgh-2017-000466.supp1 Supplementary file 1

**Table 2:** Demographic and Health Surveys (DHS) and Multiple Indicator and Cluster Surveys (MICS) included in the analytical sample

Country	DHS	MICS
Bangladesh	2004	
Benin	2006; 2011/2012	
Burkina Faso	2010	2006
Burundi	2010/2011	2005
Colombia	2010	
Congo (Brazzaville)	2011/2012	
Côte d’Ivoire	2011/2012	2006
Gabon	2012	
Ghana	2008; 2014	
Guinea	2012	
Haiti	2005/2006; 2012	
Honduras	2005/2006; 2012	
Kenya	2003; 2014	
Lao People’s Democratic Republic		2011/2012
Liberia	2007; 2011; 2013	
Malawi	2010	
Mali	2001; 2006; 2012/2013	
Mozambique	2011	
Niger	2012	
Rwanda	2005; 2010	
Senegal	2006; 2012–2014	
Sierra Leone	2008; 2013	2005
Swaziland	2006–2007	2010
Tajikistan		2005
Timor-Leste	2009/2010	
Togo	2013/2014	2006
Zambia	2007; 2013/2014	
Zimbabwe	2005/2006; 2010/2011	

**Figure 1:**
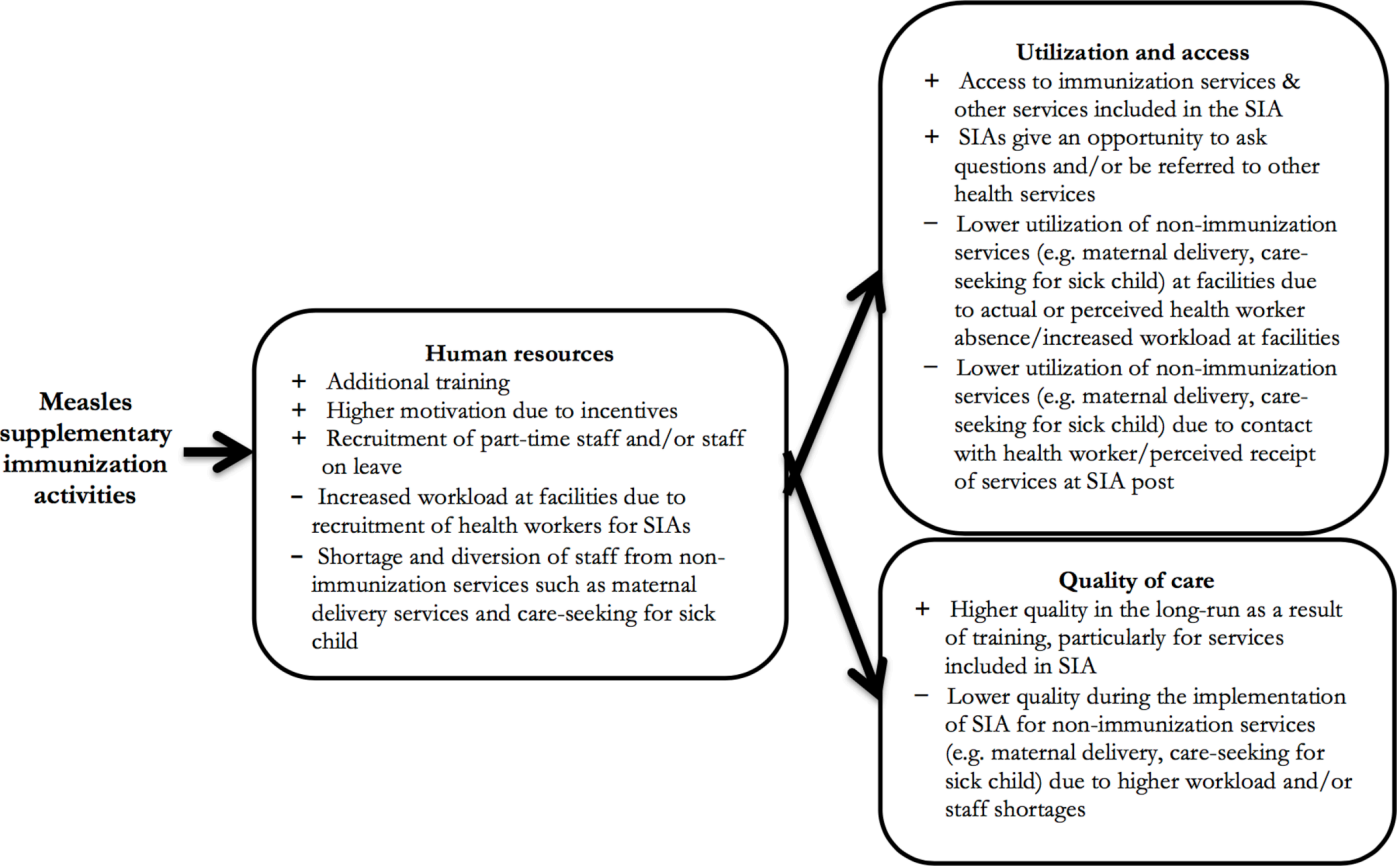
Conceptual framework of the possible effects of measles supplementary immunisation activities (SIAs) on utilisation, access, care-seeking and quality through their impact on human resources.

Based on previous research,^14 20 22 23^ we hypothesised that the main channel through which measles SIAs would affect care-seeking, utilisation and access of routine health services would be the recruitment of health workers for the campaigns (figure 1). This would result in shortage of health workers and provider absenteeism, and lead to increased workload at health facilities. As a result, the duration of the visits (outpatient care and consultations) would be shorter, the non-immunisation services could be interrupted at health facilities, and fewer patients would be able to receive care, and ultimately seek care.

Given the continuing focus on maternal and child health (MCH) services in LMICs, we focused our analysis on the utilisation of delivery and child health services. Based on data availability and plausible mechanisms of change, we included the following services in our analysis: 1) whether the woman delivered at a facility; 2) whether the newborn was weighed at birth if delivered at a facility; 3) whether the newborn received a postnatal care visit within one week of birth and 4) whether the mother sought care at a health facility if the child had fever, diarrhoea or cough within the two weeks prior to the survey. Since both surveys collect information on the type of provider from which care was sought, we were able to exclude providers that were unlikely to be affected by SIAs (eg, traditional healers, pharmacies and other non-formal providers). The full list of indicators and data availability is presented in table 3. While we did not expect to see an impact on facility delivery during SIA implementation, we hypothesised that individuals would be less likely to seek or receive postnatal and outpatient care at health facilities. We hypothesise that SIAs influence the decision to seek care at health facilities and therefore utilisation of health services. We assume that caregivers are aware of SIAs and their recruitment of health workers. Prior to the start of SIAs, countries are required to conduct social mobilisation campaigns and advertise the campaigns.^6^ Some countries even include door-to-door registration for SIAs (eg, Bangladesh),^33^ which would substantially increase awareness of campaigns. Moreover, since SIAs are typically stationed at public places (such as schools, bus depots, markets), if individuals see health workers at these posts, they may assume that they would not be present at the health facility to provide services. In addition, due to higher workload at facilities, we hypothesised that the quality of care would decline as a result of fewer available health workers and providers would skip non-essential steps, such as weighing the newborn at birth.

**Table 3:** Select maternal and child health services captured by the Demographic and Health Surveys (DHS) and the Multiple Indicator Cluster Surveys (MICS) and included in the analysis

	Domain	Indicators	DHS		MICS	
			Availability	Date	Availability	Date
Maternal care	Delivery	Delivered at a facility	✓ for births occurring in the 5 years preceding the survey	Day/month/year of birth	✓ for births occurring in the 2 years preceding the survey	Day/month/year of birth
	Postnatal care	Trained attendant checked on mother after delivery	✓ for births occurring in the 5 years preceding the survey	Day/month/year of birth	✗ Not available	
Child care	Care at birth	Weighed at birth if delivered at a facility	✓ for births occurring in the 5 years preceding the survey	Day/month/year of birth	✓ for births occurring in the 2 years preceding the survey	Day/month/year of birth
	Outpatient care	Sought care if child had fever	✓ for children under the age of 5 if the child had fever 2 weeks prior to the survey	2 weeks (14 days) before day of interview	✓ for children under the age of 5 if the child had fever 2 weeks prior to the survey	2 weeks (14 days) before day of interview
		Sought care if child had diarrhoea	✓ for children under the age of 5 if the child had diarrhoea 2 weeks prior to the survey		✗ Not available	
		Sought care if child had cough	✗ Not available		✓ for children under the age of 5 if the child had cough 2 weeks prior to the survey	2 weeks (14 days) before day of interview

### Analysis

Since the exact dates of SIAs were known, we created a dummy variable equal to 1 if the outcome occurred during the SIA campaign and 0 otherwise. We conducted logistic regressions to estimate the association between each outcome and the implementation of the SIA using the following model:

(1) $$\begin{array}{l} ln\left(\frac{Pr\left(Y_{ict}=1|X\right)}{1-Pr\left(Y_{ict}=1|X\right)}\right)=\beta_{0}+\beta_{1}I_{cSIA}+\beta_{m}Month+\beta_{y}Yr\\ \;\;\;\;\;\;\;\;\;\;\;\;\;\;\;+\beta_{c}C+\beta_{cy}CYr+\beta_{cm}CMonth+\varepsilon_{ict}, \end{array}$$

where $Y_{ict}$ is the individual-level outcome (eg, institutional delivery or care-seeking for outpatient care) and $I_{cSIA}$ is a dummy variable equal to 1 if the outcome falls within the period of the measles SIA in the country (on the day of delivery or within the 2-week time period prior to the date of interview for outpatient services). Country-differences were controlled for using country-fixed effects $\left(\beta_{c}C\right)$, and we included country-year fixed effects $\left(\beta_{cy}CYr\right)$ and country-month fixed effects $\left(\beta_{cm}CMonth\right)$ to control for different year and seasonal trends across countries, respectively. SEs were clustered at the primary sampling unit to account for the DHS and MICS survey designs.

In both surveys, mothers were asked whether they sought care if their child had fever or diarrhoea in the two weeks prior to the survey. Care-seeking for a child’s cough was also reported independently in MICS surveys, while the DHS only asked about treatment for cough if the child also presented with fever. Since the date of interview was available for all respondents, we were able to establish an exact two-week time interval. If the measles SIA in the country occurred within this two-week interval, then $I_{cSIA}=1$, and 0 otherwise. To ensure that data collection was not affected by the implementation of the SIA, we carefully examined the distribution of interviews across regions and months. In most countries, the DHS and MICS surveys were conducted in all regions simultaneously over an average of four months.

We also controlled for individual-level factors that were likely to influence the decision to seek care (woman’s age, education level and wealth quintile). In addition, due to differences in access to services, utilisation/care-seeking and mode of delivery of SIAs in urban and rural areas, we included a dummy variable equal to 1 if the individual resided in an urban area, 0 otherwise.

In addition to the pooled analysis, we also examined individual effects by country for all four services. All analyses were conducted using Stata V.14.1.

### Sensitivity analysis

For outpatient care-seeking for sick child, our exposure could be misspecified in cases where the SIA did not last for the whole two-week time period prior to the interview for which health service utilisation data are recorded. This would bias our results towards the null. Although it is reasonable to assume that there is some buffer period before the SIA is implemented during which providers attend trainings and are missing from their posts, data on training duration was not available. Therefore, to check the validity of our results for utilisation of services related to child illnesses, we limited the analysis to countries in which the SIA lasted for at least seven days during the two-week interval preceding the interview.

## Results

Table 4 presents the descriptive statistics. In our sample, 63% of women delivered at a facility, among which 94% of newborns were weighed at birth in the facility. While information on postnatal care was not available from MICS surveys, almost 77% of mothers received a postnatal check within one week of delivery according to the DHS sample. Care-seeking behaviour was most common if a child had a fever, with 54% of mothers reporting seeking care at a health facility if the child had a fever within two weeks prior to the survey. Only 40% of mothers, however, sought care at a health facility if a child had diarrhoea and 27% sought care for a child’s cough. For delivery-related outcomes, approximately 3% (n=2149) of observations coincided with the dates of measles SIA implementation (SIA dummy=1 in table 4), while the share was higher for outpatient care (11% or n=1778/3975 for diarrhoea/fever and 13% or n=380 for cough) (table 4).

**Table 4:** Summary statistics of the selected indicators from the Demographic and Health Surveys (DHS) and Multiple Indicator Cluster Surveys (MICS) included in the analysis

	Delivered at a facility		Baby weighed at birth if delivered at a facility		Received postnatal care within one week of birth*		Sought treatment at a facility for a child’s fever		Sought treatment at a facility for a child’s diarrhoea*		Sought treatment at a facility for a child’s cough†	
	N=70 620		N=38 815		N=20 015		N=37 791		N=16 739		N=2883	
	n	%	n	%	n	%	n	%	n	%	n	%
Outcome	44 574	63	36 491	94	15 454	77	20 455	54	6673	40	767	27
SIA dummy=1	2149	3	1106	3	714	4	3975	11	1778	11	380	13
Urban	21 377	30	15 475	40	6837	34	12 584	33	5568	33	685	24
Mother’s level of education												
None	26 893	38	12 418	32	7108	36	7875	21	3358	20	772	27
Primary	26 624	38	14 344	37	6984	35	17 722	47	8238	49	1516	53
Secondary or higher	17 103	24	12 053	31	5923	30	12 194	32	5143	31	595	21
Wealth quintile												
Poorest	18 397	26	7130	18	5053	25	11 360	30	5395	32	570	20
Poorer	15 779	22	7795	20	4488	22	8920	24	3914	23	622	22
Middle	13 744	19	7846	20	3826	19	7574	20	3285	20	593	21
Richer	12 278	17	8066	21	3591	18	5828	15	2498	15	630	22
Richest	10 422	15	7978	21	3057	15	4109	11	1647	10	468	16

SIA dummy=1 indicates overlap with dates of SIAs.

*Only DHS surveys.

†Only MICS surveys.

SIA, supplementary immunisation activity.

### Delivery-related services

The logistic regression results for delivery-related outcomes using a pooled sample of DHS and MICS surveys are presented in table 5. Since the exact day of birth was available, the SIA dummy variable was equal to 1 if an SIA was conducted in the country on the day of birth and 0 otherwise. Our results indicate that measles SIAs did not have an impact on delivery-related outcomes. As expected, a woman’s likelihood to deliver at a facility was not associated with the implementation of measles SIAs (OR 1.01; 95% CI 0.92 to 1.11 in the unadjusted model). The same was true for the probability of the newborn being weighed at birth if delivered at a facility (OR 1.04; 95% CI 0.77 to 1.32 in the unadjusted model) and the probability of receiving a postnatal check within one week of birth (OR 0.90; 95% CI 0.73 to 1.12). Even after adjusting for individual-level covariates (eg, wealth quintile, education and residence), the relationship between SIAs and delivery-related outcomes was not statistically significant (table 5). The odds of delivering at a facility having a newborn weighed at birth, and receiving a postnatal check were higher for women with higher levels of education, from wealthier quintiles, and those residing in urban areas.

**Table 5:** Logistic regression results of the impact of measles SIAs on facility delivery, whether the newborn was weighed at birth if delivered at a facility, and whether a postnatal check occurred within one week of birth: pooled analysis of DHS and MICS surveys

	Delivered at a facility		Newborn weighed at birth if delivered at facility		Received postnatal check within one week of birth	
	Unadjusted	Adjusted	Unadjusted	Adjusted	Unadjusted	Adjusted
SIA	1.01 (0.92 to 1.11)	0.95 (0.86 to 1.06)	1.00 (0.77 to 1.32)	0.93 (0.68 to 1.27)	0.90 (0.73 to 1.12)	0.91 (0.73 to 1.14)
Urban		2.05** (1.92 to 2.20)		2.14** (1.80 to 2.54)		1.23** (1.08 to 1.40)
No education		Reference		Reference		Reference
Primary education		1.62** (1.53 to 1.71)		1.37** (1.19 to 1.57)		1.10 (0.97 to 1.24)
Secondary or higher education		3.10** (2.88 to 3.33)		2.01** (1.66 to 2.44)		1.34** (1.16 to 1.54)
First wealth quintile (poorest)		Reference		Reference		Reference
Second wealth quintile		1.63** (1.54 to 1.73)		1.12 (0.97 to 1.28)		1.15* (1.02 to 1.30)
Third wealth quintile		2.30** (2.16 to 2.45)		1.57** (1.33 to 1.86)		1.36** (1.19 to 1.56)
Fourth wealth quintile		3.52** (3.27 to 3.79)		1.79** (1.49 to 2.14)		1.78** (1.52 to 2.09)
Fifth wealth quintile (richest)		6.80** (6.16 to 7.50)		3.56** (2.70 to 4.69)		1.71** (1.41 to 2.07)
Observations	77 504	70 620	38 852	35 901	18 566	18 063

*P<0.05, **P<0.01.

OR and 95% CIs in parentheses. Pooled analysis of DHS and MICS data. Unadjusted models include month, year and country fixed effects. Adjusted models include individual-level covariates, as well as month, year and country fixed effects. SIA dummy variable=1 if measles SIA was conducted on the day of birth.

DHS, Demographic and Health Surveys; MICS, Multiple Indicator Cluster Surveys; SIA, supplementary immunisation activity.

The individual country regression results are presented in online supplementary appendix figures A2–A4. Overall, the results from the country-level regressions were consistent with the pooled analysis, with a few exceptions. We briefly describe here such exceptions (eg, Guinea, Benin, Ghana), where the findings deviated from the pooled analysis. In Guinea, for example, women were significantly more likely to deliver at a health facility if an SIA was conducted on the day of delivery compared with women who delivered on a non-SIA day (OR 2.30; 95% CI 1.08 to 4.88). Meanwhile in Benin, newborns were less likely to get weighed at birth if they were delivered at facility on the day an SIA was being conducted (OR 0.42; 95% CI 0.18 to 0.96), while in Ghana mothers were more likely to receive a postnatal care visit within one week of giving birth during the SIAs (OR 3.81; 95% CI 1.18 to 12.29).

### Outpatient services for sick child

Table 6 presents the results from the pooled analysis for outpatient care related to child illness, and online supplementary appendix figure A5 presents the results from the individual country analysis. The dependent variables were binary indicators equal to 1 if the mother sought care at a health facility if the child had fever, diarrhoea or cough within the two weeks prior to the survey. In the unadjusted models controlling for month and survey fixed effects, our results suggest that measles SIAs were not associated with changes in the odds of seeking care across all three diseases or symptoms. After adjusting for individual-level covariates, however, caregivers were significantly less likely to seek care during measles SIAs if a child had a cough (OR 0.67; 95% CI 0.48 to 0.95). Similarly to the adjusted results in table 5, mothers with higher levels of education and from higher wealth quintiles were more likely to seek care at a health facility. In addition, those residing in urban areas were more likely to seek care at health facility if a child had fever (OR 1.42; 95% CI 1.27 to 1.60), diarrhoea (OR 1.17; 95% CI 1.06 to 1.30) or cough (OR 1.68; 95% CI 1.24 to 2.28). The results from individual country regressions (see online supplementary appendix figure A5) suggest that measles SIAs had a significant impact on care-seeking behaviour in two countries. We briefly describe here these two exceptions (eg, Burundi, Mozambique), which deviated from the pooled analysis. In Burundi, the odds of seeking care at a health facility if a child had fever were 1.46 times higher during the SIA (95% CI 1.02 to 2.09), while in Mozambique the odds were lower during an SIA (OR 0.32; 95% CI 0.18 to 0.58). In addition, we also observed lower odds of seeking care if a child had a cough in Mozambique (OR 0.25; 95% CI 0.06 to 0.99).

**Table 6:** Logistic regression results of the impact of measles SIAs on care-seeking for child fever, diarrhoea and cough: pooled analysis of DHS and MICS data

	Fever		Diarrhoea		Cough	
	Unadjusted	Adjusted	Unadjusted	Adjusted	Unadjusted	Adjusted
SIA	0.97 (0.87–1.08)	0.97 (0.87 to 1.08)	1.10 (0.94 to 1.29)	1.09 (0.93 to 1.28)	0.80 (0.59 to 1.09)	0.67* (0.48 to 0.95)
Urban		1.33** (1.24 to 1.44)		1.17** (1.06 to 1.30)		2.02** (1.42 to 2.87)
Child’s age		0.94** (0.92 to 0.95)		0.96** (0.93 to 0.98)		0.94 (0.87 to 1.01)
No education		Reference		Reference		Reference
Primary education		1.22** (1.14 to 1.31)		1.22** (1.09 to 1.35)		1.03 (0.78 to 1.36)
Secondary or higher education		1.56** (1.44 to 1.69)		1.36** (1.20 to 1.54)		1.03 (0.76 to 1.39)
First wealth quintile (poorest)		Reference		Reference		Reference
Second wealth quintile		1.17** (1.09 to 1.25)		1.05 (0.95 to 1.16)		0.80 (0.59 to 1.10)
Third wealth quintile		1.32** (1.23 to 1.43)		1.08 (0.97 to 1.21)		1.04 (0.75 to 1.45)
Fourth wealth quintile		1.35** (1.24 to 1.48)		1.16* (1.01 to 1.33)		1.20 (0.88 to 1.65)
Fifth wealth quintile (richest)		1.56** (1.40 to 1.74)		1.10 (0.94 to 1.30)		1.15 (0.78 to 1.69)
Observations	37 847	37 791	16 739	16 739	3050	2883

*P< 0.05, **P<0.01.

Binary dependent variable equal to 1 if the mother sought care at a facility if the child was ill in the two weeks prior to the survey with each symptom, 0 otherwise. ORs and 95% CIs in parentheses. Pooled analysis of DHS and MICS data. Unadjusted models include month and survey fixed effects. Adjusted models include individual-level covariates, as well as month and survey fixed effects. Questions regarding care-seeking behaviour for fever are asked in both MICS and DHS surveys, while questions regarding diarrhoea are only asked in DHS surveys and questions related to care-seeking behaviour for cough are only asked in MICS surveys.

DHS, Demographic and Health Surveys; MICS, Multiple Indicator Cluster Surveys; SIA, supplementary immunisation activity.

When we limited the analysis of outpatient utilisation to countries in which the SIA lasted for at least 7 days during the two-week interval preceding the interview, we did not find any association with health service utilisation for a child’s fever, diarrhoea or cough. The results from the sensitivity analysis are presented in online supplementary appendix table A1.

## Discussion

In this paper, we explored the impact of measles SIAs on the potential utilisation of select MCH services (eg, maternal delivery and care-seeking for child health), using nationally representative household surveys from 28 LMICs in which at least one measles SIA was conducted between 2000 and 2014. To our knowledge, this is the first multicountry study to examine the association between measles SIAs and utilisation of such MCH services. Overall, our results suggest that measles SIAs did not appear to significantly influence the utilisation of delivery services and care-seeking for sick child at health facilities. Unlike Hanvoravongchai *et al*,^14^ who found some qualitative evidence of providers skipping important tasks at health facilities due to increased workloads during measles SIAs, we did not find evidence of this in our analysis in terms of whether the newborn was weighed at birth if delivered at the facility. One service for which we did find reductions in care-seeking, however, was treatment for child cough (OR 0.67; 95% CI 0.48 to 0.95 in the adjusted model).

A possible explanation for the lower likelihood of seeking care for a child’s cough during the measles SIA could be that cough is viewed as less acute than fever or diarrhoea. If caregivers perceive that services might be disrupted during an SIA, they might choose not to seek care for a symptom that in their view might not require immediate attention. To test this hypothesis, we investigated whether caregivers were less likely to seek care if a child presented with both a cough and fever—a more severe episode linked to chronic respiratory disease. The results appear to support our hypothesis, as we did not find a significant reduction in care-seeking behaviour for children who had both symptoms (OR 0.72; 95% CI 0.45 to 1.17).

While the average effects did not seem to be significant, we did find a few significant effects at the country level. In Guinea, women were significantly more likely to deliver at facilities during SIAs (OR 2.30; 95% CI 1.08 to 4.88), and in Ghana mothers were more likely to receive postnatal care (OR 3.81; 95% CI 1.18 to 12.29). We found some evidence of providers skipping certain steps in Benin, where babies were less likely to be weighed at birth if delivered at a facility on an SIA day (OR 0.42; 95% CI 0.18 to 0.96). In Burundi, our findings suggest that caregivers were more likely to seek care at a facility during measles SIAs if a child presented with fever (OR 1.46; 95% CI 1.02 to 2.09). One possible explanation for this could be that children who presented at a measles SIA post with fever were more likely to be referred to health facilities. In Mozambique, however, our results indicated that measles SIA had a negative impact on utilisation of health services for a child’s fever or cough (OR 0.32; 95% CI 0.18 to 0.58 and OR 0.25; 95% CI 0.06 to 0.99, respectively).

The main limitations of our analysis are related to the availability of data, which constrained our ability to identify the exact timing of services and determined the services for which we could investigate the impact of SIAs. Care-seeking behaviour for child illness was only asked in relation to the two weeks preceding the survey in both MICS and DHS. Although we attempted to address this by limiting the sample to countries with more precise overlap between the SIA and the date of service utilisation for child illnesses, as pointed out earlier, longer campaigns are likely to be substantially different from shorter campaigns in their intensity and health worker involvement.^6 25^ In addition, we were not able to include services, such as reproductive health services and antenatal care, in our analysis, as the surveys did not collect dates for such services. Results from other studies indicate that reductions are in fact most pronounced for antenatal care services.^25^ Moreover, we were not able to obtain information on preparation activities, including training for health workers, and thus restricted the dates of SIAs to the delivery of the campaign. Interruption of services, however, could occur before the actual start of the SIA. For example, WHO recommends that countries conduct separate trainings for all vaccination and supervisory teams 1–3 weeks before the start of the SIA for at least two whole days.^6^ Due to data constraints, we were also not able to examine the effects of measles SIAs on quality of care. While we examined the association between measles SIAs and whether the baby was weighed at birth, which served as a proxy for quality of care, we did not investigate the impact of SIAs on other measures of quality (eg, length of outpatient consultation and content of consultation). Furthermore, our analysis did not address the impact on the broader health systems but rather examined specific non-immunisation services, that is, maternal delivery and care-seeking for a sick child. However, other health services such as routine immunisation services could be most impacted by SIAs since they require a skilled workforce (eg, nurses who may deliver injections). Lastly, many subnational and rollover SIAs also occurred in the countries we studied (table 1). Subnational campaigns might take place in regions with lower immunisation coverage and poor health services, that is, in those subnational settings with the greatest need for SIAs. The impact of subnational SIAs in these regions on care-seeking and service delivery might thus be distinct (eg, stronger impact) from national observations. Therefore, our focus on national SIAs might selectively bias our analysis towards SIAs with a less strong impact on maternal delivery and care-seeking for sick child.

While several studies have noted adverse effects of measles SIAs on utilisation of routine health services,^14 24 25^ we did not find an impact of measles SIAs for the majority of maternal and child health services we examined. The main channel through which we hypothesised that measles SIAs could lead to reductions in utilisation of delivery and care-seeking for sick child services was the absence and/or increased workload of health workers at facilities. Recent evidence from the Service Delivery Indicators surveys, however, suggests that up to 40% of providers are absent from health facilities in sub-Saharan African countries, and a large portion of such absences are excused (eg, for seminars and trainings).^34–36^ It is thus possible that during campaigns, health workers who would otherwise be absent are recruited for SIAs. As a result, the availability of health workers at health facilities might not change significantly, which would explain our findings. In addition, the lack of association for delivery services could be due to the fact that different health workers perform immunisation and delivery services. Although data are not available on the type of health workers recruited for SIAs, nurses could be more involved in campaigns, while midwives remain at facilities to deliver maternal health services. Lastly, besides measles SIAs, many recurrent campaigns occurring in LMICs, including polio immunisation campaigns and Child Health Days, may also impact provision and coverage of health services.

SIAs are promoted as an integral component of measles elimination in LMICs. In this paper, we studied the impact of measles SIAs on the utilisation of select routine health services (maternal delivery, care-seeking for sick child) using existing nationally representative survey data. While other studies have found reductions in utilisation of preventive services (eg, antenatal care and reproductive health services)^24 25^ the timing of which could be delayed, evidence from this study drawing from DHS and MICS suggests that SIAs do not appear to affect care-seeking behaviours for critical conditions, such as delivery and child fever or diarrhoea. Our results, however, point to the need for further investigation of the idiosyncratic effects in each country. This would require rigorous local data collection before, during and after the implementation of the measles SIAs, including facility-level data on utilisation of health services and the number of health workers present at the facility.
