# Supplementary material for: Impact of measles supplementary immunisation activities on utilisation of maternal and child health services in low-income and middle-income countries
Source: BMJ Glob Health. 2018 May 7;3(3):e000466. doi: 10.1136/bmjgh-2017-000466 (PMC5942424; doi:10.1136/bmjgh-2017-000466)
Supplement: Supplementary file 1 [file bmjgh-2017-000466supp001.pdf]

Supplementary appendix

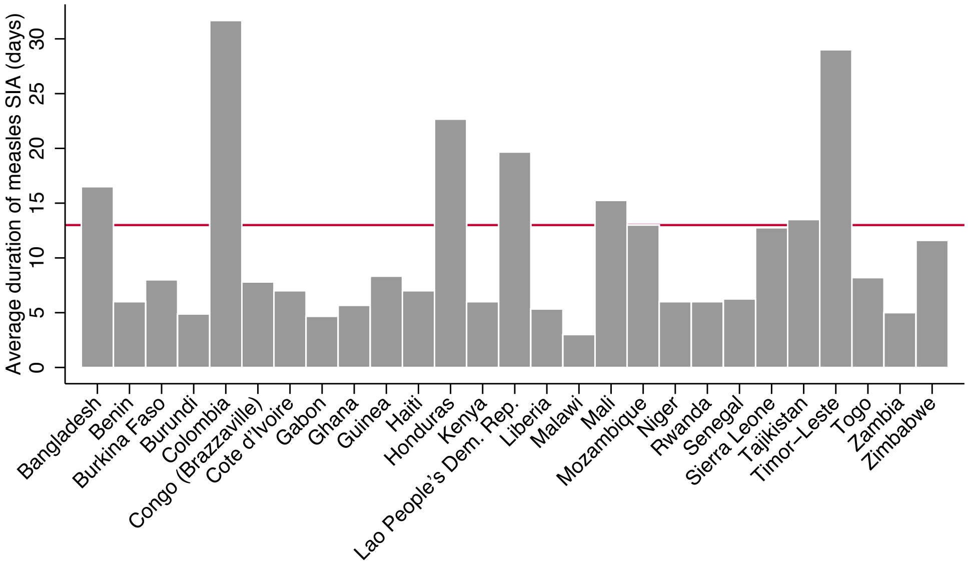

Supplementary Appendix, Figure A1. Average duration of national measles supplementary immunization activities (SIAs) included in our sample

Note: Average duration (red line) for the whole sample was 13 days and median duration was 6 days.

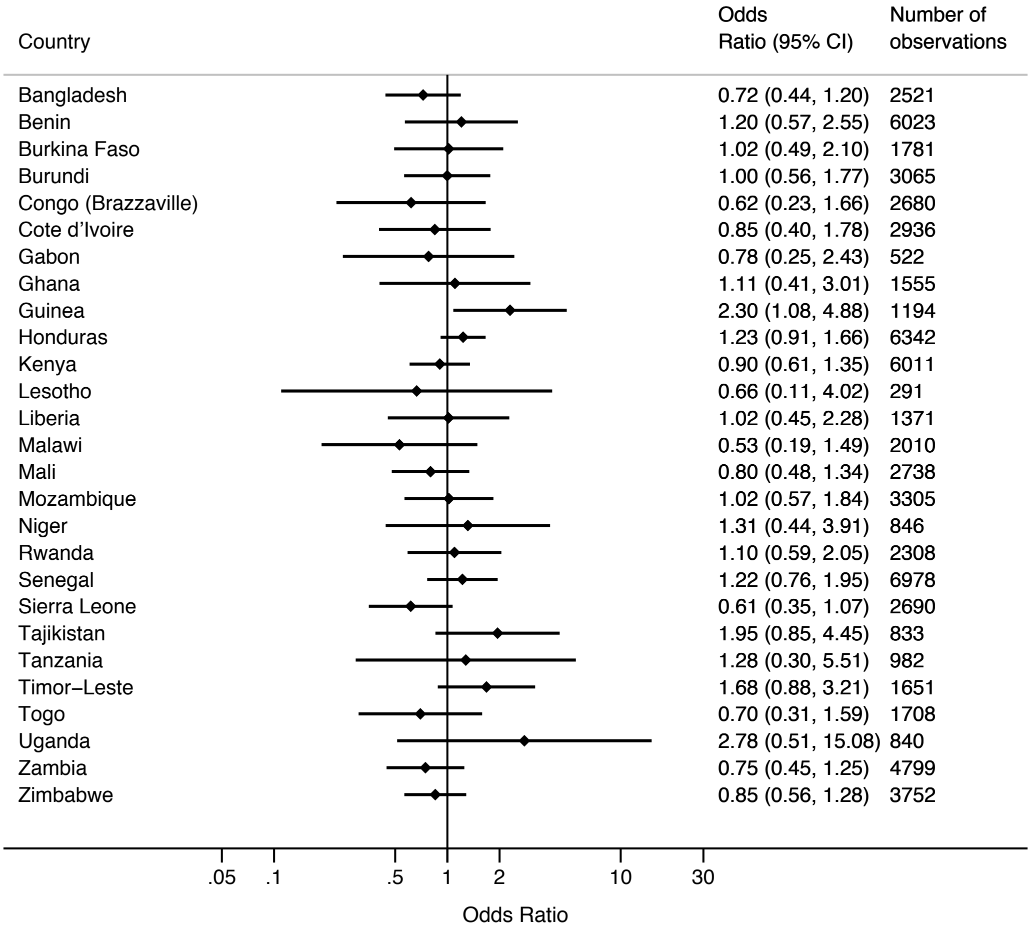

Supplementary Appendix, Figure A2. Logistic regression results of the impact of measles supplementary immunization activities (SIAs) on facility delivery: country-specific analysis

Notes: Odds ratios are presented for SIA dummy variable, which is equal to 1 if measles SIA was conducted on day of birth and 0 otherwise. The results are based on country-specific logistic regressions using the same set of covariates as the adjusted models in Table 5 in the main text. Significant results are observed for Guinea only.

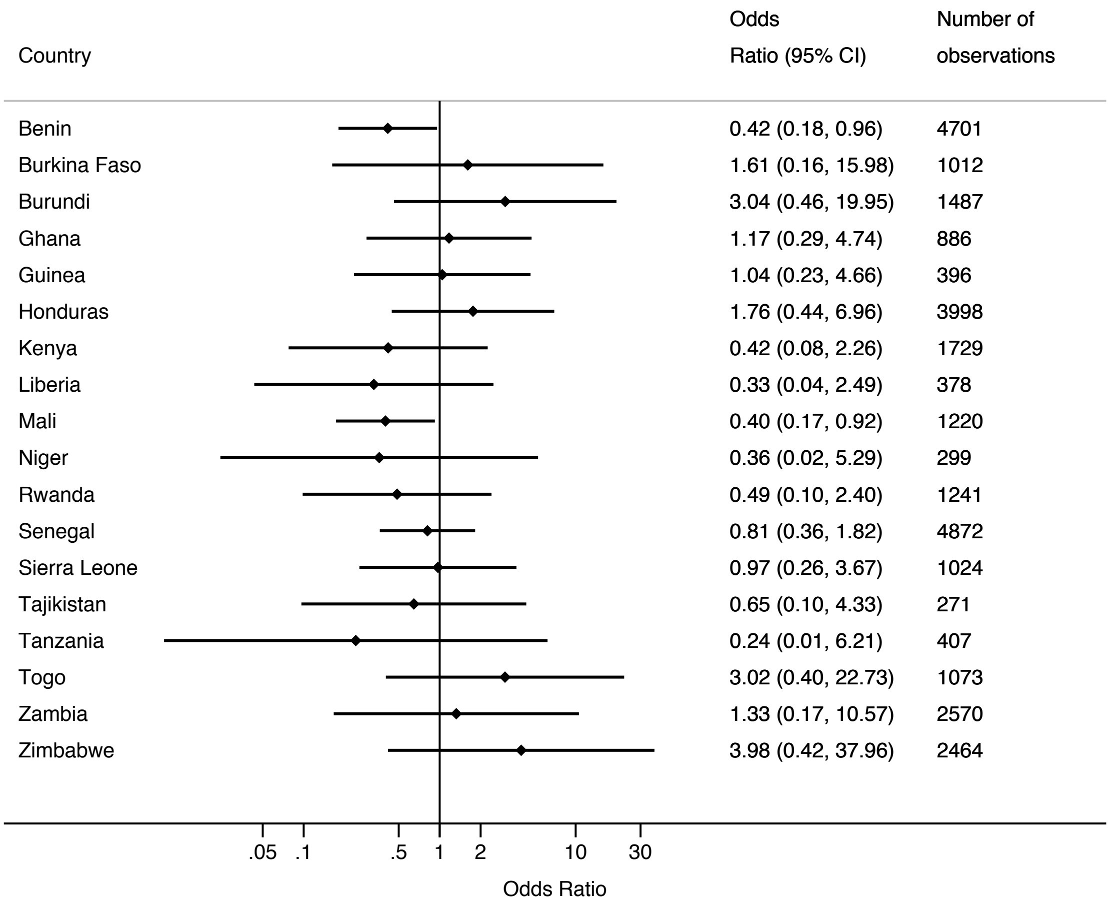

Supplementary Appendix, Figure A3. Logistic regression results of the impact of measles supplementary immunization activities (SIAs) on whether the newborn was weighed at birth if delivered at a facility: country-specific analysis

Notes: Odds ratios are presented for SIA dummy variable, which is equal to 1 if measles SIA was conducted on day of birth and 0 otherwise. The results are based on country-specific logistic regressions using the same set of covariates as the adjusted models in Table 5 in the main text. Significant results are observed for Benin and Mali only.

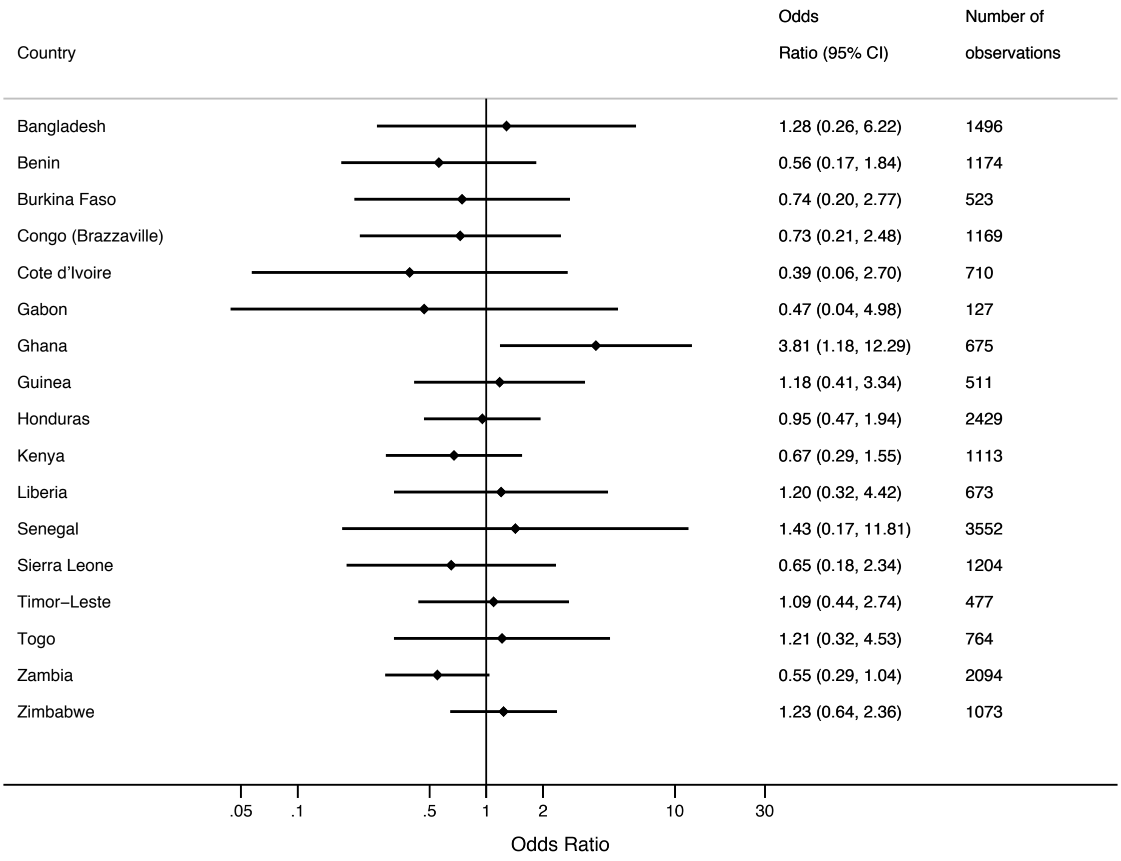

Supplementary Appendix, Figure A4. Logistic regression results of the impact of measles supplementary immunization activities (SIAs) on whether the baby received a postnatal check within one week of birth: country-specific analysis

Notes: Odds ratios are presented for SIA dummy variable, which is equal to 1 if measles SIA was conducted on day of birth and 0 otherwise. The results are based on country-specific logistic regressions using the same set of covariates as the adjusted models in Table 5 in the main text. Significant results are observed for Ghana only.

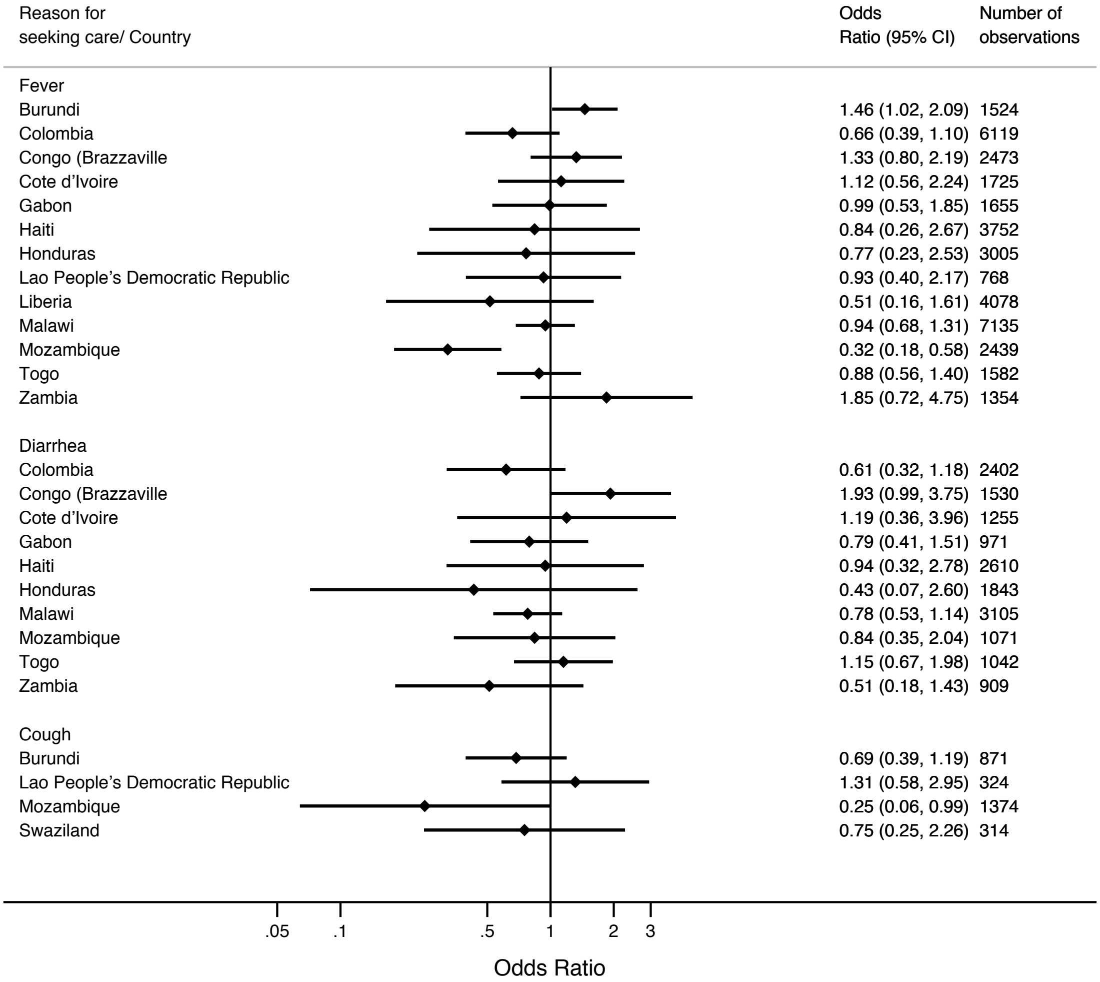

Supplementary Appendix, Figure A5. Logistic regression results of the impact of measles supplementary immunization activities (SIAs) on care seeking for child fever, diarrhea, and cough: country-specific analysis

Notes: Odds ratios are presented for SIA dummy variable, which is equal to 1 if measles SIA was conducted during the two-week time period preceding the date of interview and 0 otherwise. The results are based on country-specific logistic regressions using the same set of covariates as the adjusted models in Table 6 in the main text. Significant results are observed for Burundi and Mozambique only.

Supplementary Appendix, Table A1. Logistic regression results of the impact of measles supplementary immunization activities (SIAs) on care-seeking for child fever, diarrhea, and cough: pooled analysis of DHS and MICS data: sensitivity analysis

	Fever	Diarrhea	Cough
SIA	0.99	0.865	0.838
	(0.839 - 1.167)	(0.636 - 1.176)	(0.572 - 1.228)
Urban	1.403**	1.279**	1.337
	(1.272 - 1.547)	(1.104 - 1.481)	(0.886 - 2.016)
Child's age	0.938**	0.939**	0.915*
	(0.918 - 0.957)	(0.904 - 0.975)	(0.849 - 0.986)
No education		*ref.*	
Primary education	1.189**	1.215*	1.344
	(1.079 - 1.310)	(1.027 - 1.437)	(0.962 - 1.877)
Secondary or higher education	1.440**	1.353**	1.254
	(1.292 - 1.605)	(1.121 - 1.633)	(0.910 - 1.728)
1st wealth quintile (poorest)			
2nd wealth quintile	1.195**	1.164*	0.713
	(1.090 - 1.310)	(1.010 - 1.342)	(0.503 - 1.011)
3rd wealth quintile	1.345**	1.169	0.903
	(1.208 - 1.497)	(0.983 - 1.389)	(0.635 - 1.284)
4th wealth quintile	1.327**	1.291*	1.091
	(1.170 - 1.507)	(1.047 - 1.594)	(0.762 - 1.564)
5th wealth quintile (richest)	1.425**	1.258	1.057
	(1.222 - 1.661)	(0.973 - 1.625)	(0.684 - 1.632)
Observations	19,927	8,299	1,509

Notes: Sample limited to countries with SIAs lasting for at least 7 days during the two-week time period. Binary dependent variable equal to 1 if the mother sought care at a facility if the child was ill in the two weeks prior to the survey with each symptom, 0 otherwise. Odds ratios and 95% CI in parentheses. * indicates significant level of $p < 0.05$; ** indicates significance level of $p < 0.01$.
